# Growth with high planktonic biomass in *Shewanella oneidensis* fuel cells

**DOI:** 10.1111/j.1574-6968.2007.00964.x

**Published:** 2007-11-08

**Authors:** Martin Lanthier, Kelvin B Gregory, Derek R Lovley

**Affiliations:** 1 Eastern Cereal and Oilseed Research Center, Agriculture and Agri-Food Canada Ottawa, ON, Canada; 2 Department of Civil and Environmental Engineering, Carnegie Mellon University Pittsburgh, PA, USA; 3 Department of Microbiology, University of Massachusetts Amherst, MA, USA

**Keywords:** *Shewanella*, microbial fuel cell, biofilm, anaerobic respiration

## Abstract

*Shewanella oneidensis* MR-1 grew for over 50 days in microbial fuel cells, incompletely oxidizing lactate to acetate with high recovery of the electrons derived from this reaction as electricity. Electricity was produced with lactate or hydrogen and current was comparable to that of electricigens which completely oxidize organic substrates. However, unlike fuel cells with previously described electricigens, in which cells are primarily attached to the anode, at least as many of the *S. oneidensis* cells were planktonic as were attached to the anode. These results demonstrate that *S. oneidensis* may conserve energy for growth with an electrode serving as an electron acceptor and suggest that multiple strategies for electron transfer to fuel cell anodes exist.

## Introduction

There is a compelling need to identify pure cultures that can serve as appropriate models for electron transfer to the anodes of microbial fuel cells because little is known about the physiology of this process. *Shewanella* species were the first organisms proposed to transfer electrons to the surface of electrodes via electron-transfer proteins (Kim *et al*., 1999a, b, 1999). This, coupled with the relative ease in growing these organisms, has led to their common use as model organisms for the study of microbial fuel cells (Kim *et al*., 2002; Park & Zeikus, 2002; Gorby *et al*., 2006; Ringeisen *et al*., 2006; Biffinger *et al*., 2007; Cho & Ellington, 2007).

However, little is known about the physiology of *Shewanella* species growing with anodes serving as the sole electron acceptor. Most studies (Kim *et al*., 2002; Park & Zeikus, 2002; Cho & Ellington, 2007) have evaluated electricity production in short-term studies with cell suspensions. Cell suspension studies do not give appropriate information on the long-term functioning of microbial fuel cells that is necessary for applications (Shukla *et al*., 2004; Lovley, 2006). As previously discussed (Lovley, 2006), in the one previous study designed to evaluate *Shewanella* growth with an electrode serving as the electron acceptor (Kim *et al*., 1999a, b, 1999), growth was not directly coupled to electricity production and a low percentage (<10%) of the electrons available in the electron donor (lactate) was recovered as current. The possibility that the cultures might have been contaminated (Kim *et al*., 2002) further complicates analysis of the data. In a more recent study, which was designed to optimize power output of a growing culture of *Shewanella oneidensis*, electron recovery as current remained <10% (Ringeisen *et al*., 2006).

In order to learn more about the long-term sustainability of *Shewanella*-based microbial fuel cells, the growth of the most commonly studied strain of the *Shewanella* genus, *S. oneidensis* MR-1, was investigated. The results demonstrate that mechanisms for growth and electron transfer with electrodes serving as the electron acceptor in *S. oneidensis* are different than the patterns observed in electricigens, microorganisms that completely oxidize organic fuels with direct electron transfer to the anode surface.

## Experimental

### Growth conditions and microbial fuel cells

*Shewanella oneidensis* strain MR-1 (ATCC 7005500), was grown in anaerobic, freshwater, lactate (20 mM)-Fe(III) citrate (50 mM) medium (FW medium) as described previously (Lovley *et al*., 1989) with the exception that the three amino acids typically added to the medium (l-arginine, l-glutamine, and dl-serine) were deleted and the medium was amended with 0.05% (w/v) yeast extract. The cells from a 100 mL culture near the end of log phase were collected by centrifugation and resuspended in FW medium (5 mL) without yeast extract or Fe(III) citrate. These cells (3.7 mg of total protein) were inoculated into the anode chamber of previously described (Bond & Lovley, 2003) fuel cells in which graphite sticks (surface area 61.2 cm^2^) serve as the anode and cathode. The electrical connection between the anode and cathode included a resistor of 560 Ω (Bond & Lovley, 2003). Current was measured hourly with a Keithley Model 2700 Digital Multimeter (Keithley Instruments, Cleveland, OH). The anode chamber contained 225 mL of FW medium described above or ‘defined FW medium’ in which the yeast extract was omitted and substituted with l-arginine (22 mg L^−1^), l-glutamine (22 mg L^−1^), and dl-serine (44 mg L^−1^). The initial lactate concentration was 10 mM. The cathode chamber contained 225 mL of FW medium without yeast extract, amino acids, or Fe(III). The anode chamber was continuously bubbled with N_2_/CO_2_ (80 : 20) and the cathode chamber was continuously bubbled with air. All incubations were at 30 °C.

### Protein quantification

Total protein content from planktonic cells in the anode chambers was quantified after each exchange of the medium and after termination of the experiments. Protein concentration was determined on centrifuged cell pellets and on biofilm scraped from electrodes with sterile razorblades by the bicinchoninic acid method with bovine serum albumin as a standard (Smith *et al*., 1985).

### Microscopy

Cells associated with the anodes were stained with the BacLight Live/Dead viability kit (Invitrogen–Molecular Probes Inc., Eugene, OR), as described previously (Korber *et al*., 1996) before examination with confocal laser scanning microscopy (CLSM). Samples were examined on a Zeiss Axiovert LSM 510 Meta Confocal System equipped with a 63 × Zeiss plan-apochromat oil immersion objective (numerical aperture of 1.4) and a Meta detector (Carl Zeiss MicroImaging Inc., Thornwood, NY). The confocal microscope was equipped with an argon laser (lines at 458, 477, 488 and 514 nm; 25 mW total), and two HeNe lasers (543 nm, 1 mW; 633 nm, 5 mW), krypton–argon dual laser (488 and 568 nm) and a diode laser (638 nm). Representative three-dimensional scans of biofilm sections on the electrodes were taken, and displayed as ortho view. Images were averaged by Kalman filtration with eight running scans per image (Manz *et al*., 1999). The acquisition software was lsm 510 meta, version 3.2 SP2. The ratio of live/dead cells on the anodes was determined by quantifying the ratio of dead cells (red) and live cells (green) from confocal images as described previously (Lanthier *et al*., 2005). The imagej software v1.31 (US National Institutes of Health, Bethesda, MD, http://rsb.info.nih.gov/ij/) was used for image analysis. Anodes sacrificed for SEM were prepared as described previously (Bond & Lovley, 2003) except that the whole electrode was chemically dehydrated with hexamethyldisilazane.

## Results and discussion

### Current production

**Fig. 1:**
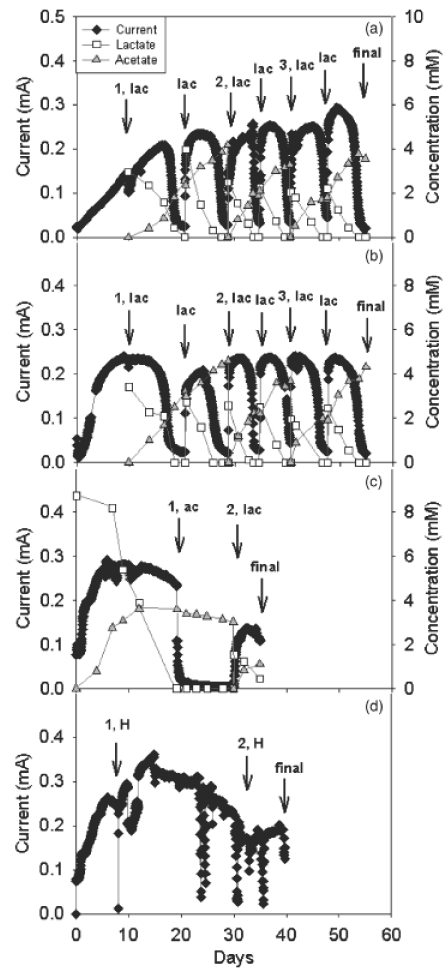
Current production, lactate consumption and acetate accumulation by *Shewanella oneidensis* MR-1 growing with lactate as an electron donor and an anode as the sole terminal electron acceptor in (a) defined FW medium; (b) FW medium supplemented with 0.05% yeast extract. Fuel cells fed with acetate in defined FW medium (c) or hydrogen in defined FW medium (d) were first started with lactate as an electron donor, and medium was exchanged with the appropriate electron donor when current production was steady. First (1), second (2) or third (3) medium exchange in the anode chamber was accompanied by addition of 3 mM lactate (lac), 4 mM acetate (ac), or bubbling with hydrogen (as H_2_/CO_2_/N_2_) (H), as noted. Sampling of medium and electrode at the end of experiment was dubbed ‘final’ (final). The medium in fuel cell C was only exchanged twice.

With either the yeast extract-amended or defined medium, current production increased over time to a maximum of 0.2–0.3 mA (Fig. 1a and b). Maximum current was typically reached earlier in the medium containing yeast extract than the defined medium, but there was no consistent difference in the maximum current between the two medium types (Fig. 1). The anode chambers were turbid with planktonic cells and higher turbidity was observed with yeast extract-amended medium (see biomass quantification by protein assay in the next section). The medium in the anode chambers was exchanged by removing the medium and planktonic cells (but retaining anode-associated cells) and replacing with new FW medium (Bond & Lovley, 2003). After addition of 3 mM lactate, current production resumed at levels comparable (0.2–0.3 mA) to those before exchanging the medium within 12 h in defined medium and within 3 h in medium containing yeast extract. This observation was consistent after each exchange of the medium (Fig. 1a and b).

**Fig. 2:**
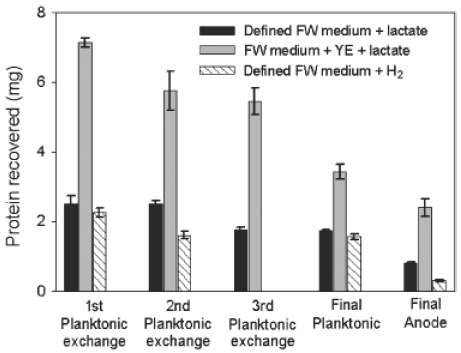
Protein content of the medium in the anode chamber and on the anode of fuel cells inoculated with *Shewanella oneidensis* MR-1. Protein from planktonic cells was determined after each exchange of the medium. Protein from both planktonic and anode associated cells was determined after termination of the experiment. All fuel cells contained FW medium supplemented with lactate, except the fuel cell with hydrogen, which was supplemented with lactate and no hydrogen until the first medium exchange, and then bubbled with hydrogen without lactate. No third medium exchanged was performed on the fuel cell with defined medium and hydrogen.

Addition of yeast extract did not increase current production, but increased protein biomass both in suspension and on the anode (Fig. 2). Current was not observed in the absence of cells (data not shown), or in the presence of 4 mM acetate (Fig. 1c), an electron donor not utilized by *S. oneidensis* under anaerobic conditions (Lovley *et al*., 1992). When the acetate medium was exchanged with medium containing lactate, power production resumed, but at a somewhat lower initial rate (Fig. 1c). This is presumably due to the inability of cells to maintain as active a population during the incubation with acetate.

In order to ensure that the current production in the presence of cells and lactate was not limited by reactions at the cathode, similar studies were also conducted in which the anode was poised with a potentiostat at +300 mV (vs. Ag/AgCl reference) as described previously (Bond & Lovley, 2003). However, sustained power production in the poised system was no higher than in fuel cells (data not shown). Therefore, all remaining studies were conducted as fuel cells.

### Growth and electron recovery

The protein content from planktonic cells in the anode chambers was quantified after each exchange of the medium and after termination of the experiments (as indicated in Fig. 1). Protein on the anode was determined only after termination of the experiments (Fig. 1). There were substantial amounts of planktonic protein biomass in the anode chambers with higher quantities of planktonic protein biomass in the yeast-extract amended medium than the defined medium (Fig. 2). Planktonic cells became abundant each time the anode medium was exchanged, as indicated by the return of visible turbidity and measured by high planktonic cell protein after each medium exchange (Fig. 2). Planktonic protein biomass declined with each medium exchange accompanied by lactate amendment. The decrease in planktonic protein biomass over the course of the experiment was not as large in defined medium as in yeast extract amended medium. After multiple medium replacements (Fig. 1a, b and d), the anodes were removed from the anode chamber and analyzed for protein. There was less attached protein biomass than planktonic protein biomass at the termination of the experiment (Fig. 2). There was more protein biomass on the anodes from yeast extract medium than defined medium.

Growth of MR-1 with the anode serving as the sole electron acceptor was further evaluated by inoculating a fuel cell containing defined FW medium and 10 mM lactate with a small inoculum of cells (0.04 mg protein). After 10 days, current was produced at a level equivalent to that observed with the larger inocula shown in Fig. 1. After 14 days, the total protein in the anode chamber and on the anode surface was 2.29 mg (anode: 0.28 mg). These results demonstrate that *S. oneidensis* may conserve energy to support growth using the anode in a fuel cell as the sole terminal electron acceptor.

**Fig. 3:**
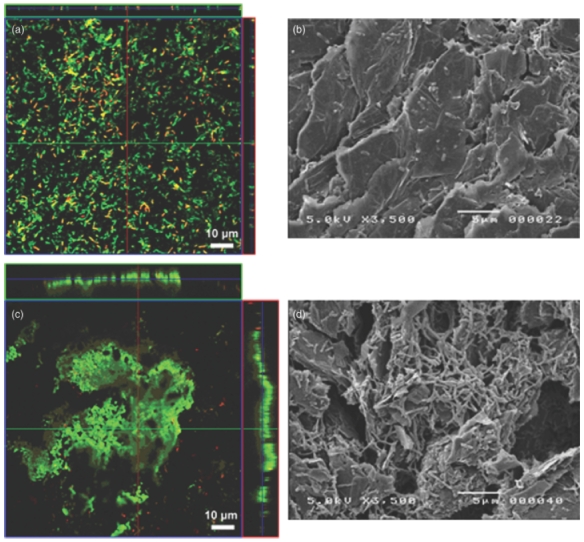
Confocal (a, c) and SEM (b, d) pictures of the biofilm growing on anodes of fuel cells inoculated with *Shewanella oneidensis* MR-1. Fuel cells were fed with FW medium supplemented with lactate and (a, b) amino acids; (c, d) yeast extract. Anodes for CLSM examination were directly stained with the BacLight Live/Dead kit (Korber *et al*., 1996). Live cells appear green and dead cells red.

Growth on the anodes was also apparent with CLSM and SEM (Fig. 3). Cells were scattered over the surface of the anodes from fuel cells with defined medium (Fig. 3a and b). There was greater coverage of the anodes from fuel cells with yeast extract medium, but there were still substantial portions of the anode that were not colonized with cells and in no instance were thick biofilms of multiple stacked cells observed (Fig. 3c and d). CLSM images indicate a higher density of live cells on the anode recovered from the fuel cell amended with yeast extract (97.0±3.5%; *n* = 7) than in defined medium (75.2±7.2%; *n* = 7), further suggesting that the yeast extract-amended medium was a superior medium for growth in fuel cells. These anaerobic anode biofilms differ significantly from the much more extensive biofilms that *S. oneidensis* MR-1 forms when grown in aerobic flow-through cells with rich medium (Thormann *et al*., 2004).

Analysis of the stoichiometry of lactate metabolism and electricity production demonstrated that lactate was consumed over time with an approximate (1 : 1) stoichiometric accumulation of acetate (Fig. 1a and b). The medium was exchanged after every second addition of lactate in the microbial fuel cells. For the first additions of lactate after the medium was exchanged, the accumulation of acetate from lactate was 86.8±1.6% (mean±SD; *n* = 3) in the defined medium, whereas in the yeast extract-amended medium the recovery was 94.9±5.4%. These results show that lactate was partially oxidized by MR-1 according to the reaction 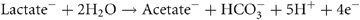
which is consistent with previous studies (Lovley *et al*., 1989, 1992).

The coulombic yield based on partial lactate oxidation (4 e^−^ mol^−1^ lactate) was 55.9±12.9% (*n* = 3) in defined medium and 56.2±14.8% for the medium amended with yeast extract. In this case, a coulombic yield less than 100% reflects the fraction of electrons obtained from partial lactate oxidation which were diverted to cell synthesis, rather than electricity production, during growth of MR-1 on the anode. Similar electron recovery was observed with MR-1 during growth on nitrate (Middleton *et al*., 2003) and are typical of other bacteria which exhibit anaerobic nonfermentative respiration and growth (Löffler *et al*., 1999).

In order to evaluate the potential for electricity production with hydrogen as the electron donor, cells were initially inoculated into an anode chamber containing defined lactate medium and once current production was established, the medium was replaced with lactate-free medium and continuously bubbled with a mixture of H_2_/CO_2_/N_2_ (7 : 10 : 83). Current production was comparable to that with lactate as the electron donor (Fig. 1d). As in lactate cultures, there was visible turbidity in the anode chamber, corresponding with the recovery of cell protein in the medium (Fig. 2). Turbidity decreased after the media was exchanged with lactate-free medium, but remained stable each time the medium was exchanged afterward (Fig. 2). There was less protein biomass attached to the anode than planktonic protein biomass, consistent with the results with lactate as the fuel (Fig. 2).

## Conclusion

These results further demonstrate the diversity of strategies that microorganisms may employ for growth with electrodes serving as the electron acceptor. For example, in previous studies with other pure cultures (Bond & Lovley, 2003, 2005; Chaudhuri & Lovley, 2003; Holmes *et al*., 2004a, b), the electricity-producing microorganisms were predominately attached to the anode surface. In contrast, most of the cellular protein in the *S. oneidensis* fuel cells presented in this study were planktonic. *Shewanella* species are capable of producing electron shuttles, which permit them to reduce insoluble Fe(III) oxides without direct cell-electron acceptor contact (Newman & Kolter, 2000; Nevin & Lovley, 2002a, b; Lies *et al*., 2005). Although these electron shuttles are generally considered to be small, soluble molecules, they might also be comprised of the membrane vesicles containing redox proteins that are known to be released from gram-negative microorganisms (Krause *et al*., 1996; Schooling & Beveridge, 2006). It seems likely that electron shuttle production permits *S. oneidensis* to oxidize lactate or hydrogen without being attached to the anode surface as electrochemical analyses suggest electron-shuttling vs. direct electron transfer in *Shewanella*-based microbial fuel cells (Marsili *et al*., 2007). For some organisms, such as *Geobacter* species, which do not produce electron shuttles (Nevin & Lovley, 2000; Bond & Lovley, 2003) close association of the cells with the anode is likely to be essential for electron transfer (Bond & Lovley, 2003; Reguera *et al*., 2006). However, even *Geothrix fermentans*, which produces an electron shuttle (Nevin & Lovley, 2002a, b) remains affixed to the anode surface in microbial fuel cells (Bond & Lovley, 2005).

Incomplete oxidation of lactate to acetate coupled to electron transfer to electrodes similar to that reported here for *S. oneidensis*, was observed previously in *Geothrix fermentans* (Bond & Lovley, 2005). However, *G. fermentans* subsequently oxidized the acetate whereas *S. oneidensis* cannot. This is an important consideration because this means that only a third of the electrons available in the initial fuel is converted to electricity.

Although the ability of *Shewanella* species to produce electricity from lactate has been noted many times (Kim *et al*., 1999a, b, 1999, 2002; Ringeisen *et al*., 2006; Biffinger *et al*., 2007; Cho & Ellington, 2007), the results presented here demonstrate for the first time that a substantial proportion of the electrons derived from lactate oxidation by *S. oneidensis* can be recovered as electrons in a microbial fuel cell. This is an important consideration because electron recoveries of <1% (Kim *et al*., 2002) and 10% (Ringeisen *et al*., 2006), are difficult to reconcile with the concept that *Shewanella* species may use electrodes as a respiratory electron acceptor.

The possibility of producing electricity with microbial cultures has been known for a long time, the study of self-sustaining microbial fuel cells in which microorganisms, known as electricigens, conserve energy to support growth from electron transfer to electrodes is in its infancy (Lovley, 2006). To date, each pure culture study with electricigens has demonstrated that the particular organism under investigation has properties in fuel cells that are significantly different than previously studied organisms (Bond *et al*., 2002; Bond & Lovley, 2003, 2005; Chaudhuri & Lovley, 2003; Holmes *et al*., 2004a, b; Biffinger *et al*., 2007). The finding that *S. oneidensis* offers yet another variation on growth on electrodes emphasizes the need to further evaluate the diversity of microorganisms capable of respiration with electrodes in order to realize the full range of microbial strategies that can be potentially exploited for the optimization of microbial fuel cells.

## Statement

OnlineOpen articles are made available in accordance with the terms of the Creative Commons Deed, Attribution 2.5 (further details available from http://www.creativecommons.org), which allows Open Access dissemination of the article, but does not permit commercial exploitation or the creation of derivative works.
